# Lifelong Impact of Variations in Maternal Care on Dendritic Structure and Function of Cortical Layer 2/3 Pyramidal Neurons in Rat Offspring

**DOI:** 10.1371/journal.pone.0005167

**Published:** 2009-04-09

**Authors:** Laura A. Smit-Rigter, Danielle L. Champagne, Johannes A. van Hooft

**Affiliations:** 1 Swammerdam Institute for Life Sciences, Center for Neuroscience, University of Amsterdam, Amsterdam, The Netherlands; 2 Department of Medical Pharmacology, Leiden University Medical Center and the Leiden/Amsterdam Center for Drug Research, Leiden, The Netherlands; Universidade Federal do Rio de Janeiro (UFRJ), Instituto de Biofísica da UFRJ, Brazil

## Abstract

Maternal licking and grooming (LG) exerts profound influence on hippocampal development and function in the offspring. However, little information is available on the effects of variations in maternal care on other brain regions. Here we examined the effects of variation in the frequency of maternal LG on morphological and electrophysiological properties of layer 2/3 pyramidal neurons in the somatosensory cortex in adult offspring. Compared to low LG offspring, high LG offspring displayed decreased dendritic complexity, reduced spine density and decreased amplitude of spontaneous postsynaptic currents. These changes were accompanied by higher levels of reelin expression in offspring of high LG mothers. Taken together, these findings suggest that differential amount of naturally-occurring variations in maternal LG is associated with enduring changes in dendritic morphology and synaptic function in layer 2/3 pyramidal neurons of the somatosensory cortex.

## Introduction

Exposure to adverse childhood events is linked to increased risk of developing psychopathologies in later life in addition to behavioral and cognitive impairments [1]. In the rodent maternal care model, naturally-occurring variations in the amount of maternal licking and grooming (LG) provided by the dam during the first week of life are associated with individual differences in stress responsiveness, emotionality and cognitive functioning in adult offspring. Pups that receive a high frequency of LG exhibit reduced fearfulness and dampened responsiveness to stress in adulthood [2]. Furthermore, when compared to low LG, high LG offspring shows enhanced synaptogenesis and neuronal survival in the hippocampus which is in agreement with the greater spatial learning and memory performances observed in the latter [3], [4]. Remarkably, the results of cross-fostering studies provided strong evidence for a causal relationship between quality of maternal care and development of differential phenotypes in offspring as well as transmission of maternal behavior across generations [4]–[6]. Recent evidence demonstrated impact of maternal care on epigenetic modulation of gene expression, such as the glucocorticoid receptor (GR), as part of the mechanisms underlying long-lasting effects of differential maternal care on adult phenotypes [7]–[9]. Interestingly, this phenomenon has also been shown to occur in humans with history of early-life stress [10], [11].

Recently, a study from our group showed that variations in maternal LG influenced morphological and functional (hippocampal-dependent learning) aspects of CA1 hippocampal neurons [12], suggesting a strong influence of maternal care on hippocampal development and ability to induce experience-dependent plasticity in limbic areas through variations in LG. Specifically, it was shown that hippocampal CA1 pyramidal neurons of adult offspring from high LG mothers exhibited longer dendrites and increased spine density as compared to offspring of low LG mothers [12].

Other studies have shown that mRNA levels of reelin, a glycoprotein which plays an important role during cortical and hippocampal development and with a promotor which is highly sensitive to epigenetic modulation, are elevated in the hippocampus of high LG rats [13]–[17]. In addition to these results, we recently found that in the postnatal cortex, reelin regulates dendritic arborization of layer 2/3 pyramidal neurons [18]. Taken this into account, we sought to determine whether dendritic morphology and function of cortical layer 2/3 pyramidal neurons and reelin levels vary in response to naturally-occurring variations in maternal care.

## Results and Discussion

**Figure 1:**
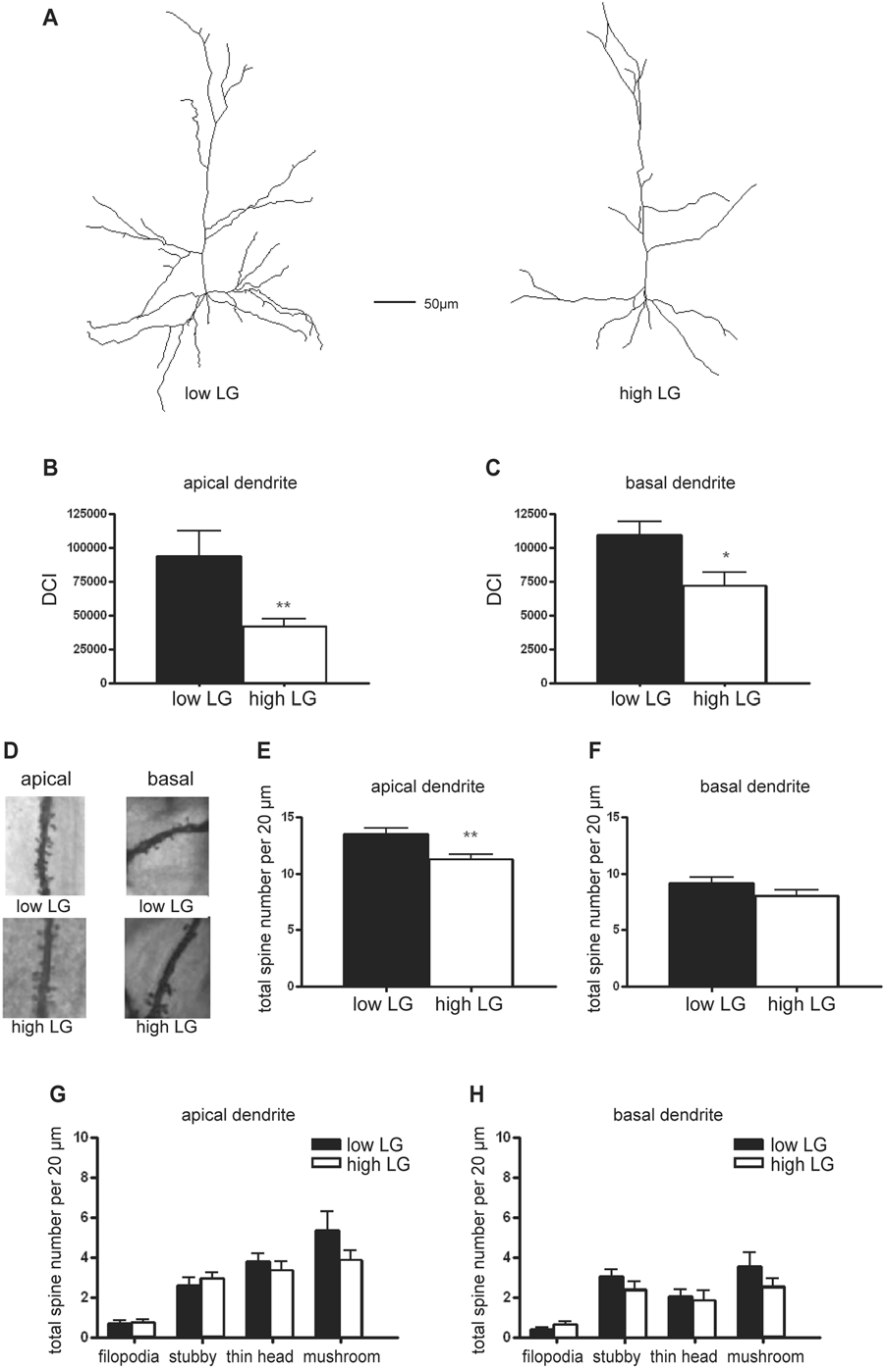
Maternal LG has a lifelong effect on dendritic complexity and total spine density in the cortex. (*A*) Representative examples of reconstructed cortical layer 2/3 pyramidal neurons in the somatosensory cortex of male adult high and low LG rats. (*B,C*) Dendritic complexity index calculated for the apical (*B*) and basal (*C*) dendrites of layer 2/3 pyramidal neurons of high and low LG rats. (*D*) Representative images of Golgi-impregnated segments from apical and basal dendrites of layer 2/3 pyramidal neurons in the somatosensory cortex of male adult high and low LG rats. (*E,F*) Average total spine distribution per 20 µm on the apical (*E*) and basal (*F*) dendrites in high and low LG rats. *(G,H)* Average spine distribution per 20 µm for each spine subtype on the apical *(G)* and basal *(H)* dendrites of high and low LG rats. Data are expressed as the mean±SEM. Asterisks indicate significant differences (* p<0.05, ** p<0.01).

The dendritic morphology of layer 2/3 pyramidal neurons in the somatosensory cortex was studied in sets of Golgi-stained sections obtained from high- and low LG adult rats used in a previous study [12]. Generally, the dendritic tree of pyramidal neurons in high LG rats displayed a slender morphology, with only a few branches over the length of the dendritic tree as compared to low LG rats (Figure 1A). The total dendritic length of the dendritic tree of layer 2/3 pyramidal neurons from high LG rats was significantly lower than that of low LG rats (Table 1.) The differences in morphology were quantified using the dendritic complexity index (DCI) [19]. In high LG rats, DCI of the apical dendrites of layer 2/3 pyramidal neurons was reduced as compared to that of the low LG rats (p = 0.008, high LG: *n* = 24, low LG: *n* = 21, Figure 1B). Similar results were obtained for the basal dendrites (p = 0.013, Figure 1C).

**Table 1:** Maternal LG has an effect on various components of the dendritic complexity index.

	apical		basal	
	high LG	Low LG	high LG	Low LG
**Total arbor length (µm)**	862±54.0 *	1106±78.2	781.2±48.3 **	1008.9±53.1
**Branch tip order**	35.3±3.2 **	61.4±7.6	20.3±2.0 *	28.3±2.3
**Branch tip number**	8.9±0.5 **	12.3±1.0	10.8±0.5 **	13.1±0.7
**Primary dendrite number**	1±0	1±0	3.8±0.1	4.0±0.2

To calculate the dendritic complexity index for the apical and basal dendrites of cortical layer 2/3 pyramidal neurons of high LG and low LG rats total arbor length, branch tip order, branch tip number and primary dendrite number were analyzed. Data are expressed as the mean±SEM. Asterisks indicate significant differences (* p<0.05, ** p<0.01).

We also analysed the density of dendritic spines on both the apical and basal dendritic trees. Spines were classified according to Harris et al. [20] as filopodia, stubby, thin head or mushroom (Figure 1D). The total spine density on the apical dendrites of layer 2/3 pyramidal neurons in the somatosensory cortex was reduced in high LG rats as compared to low LG rats (p = 0.004, high LG *n* = 28, low LG *n* = 27, Figure 1E), without any difference in the distribution between the different types of spines (Figure 1G). No significant changes in spine density were detected for the basal dendrites (Figure 1F and H). The effects of variations in maternal care on the dendritic maturation of layer 2/3 pyramidal neurons in the cortex are opposite to our previous findings in hippocampal CA1 pyramidal neurons, which displayed longer total dendritic length and increased number of total spines in high LG rats [12]. The reason underlying such discrepancy is unclear. A variety of paradigms used to investigate the effects of environmental stimuli such as maternal separation and environmental enrichment have shown similar differential effects on dendritic morphology, depending on the context, time window, and duration of the environmental stimuli [21]–[23]. A variety of factors such as growth factors, hormones, neurotransmitters, and immediate early genes are released or induced, which in turn exert their effects on dendritic branching and spine maturation [24]–[26]. Taken together these findings suggest that the effects of environmental stimuli on dendritic morphology, such as those described above, appear to be mostly region-specific, a phenomenon that is extended to include the maternal care model (present study).

**Figure 2:**
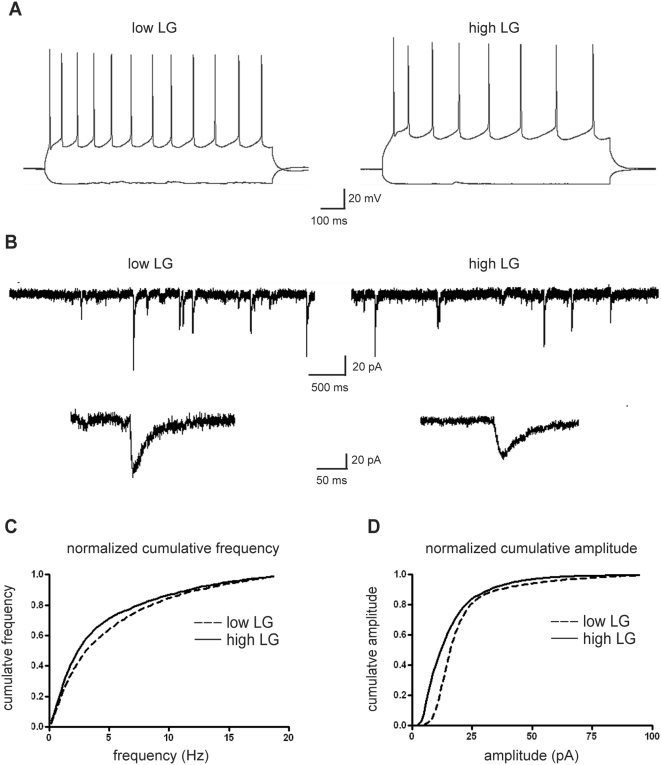
Maternal LG affects the amplitude of spontaneous postsynaptic currents in cortical layer 2/3 pyramidal neurons. (*A,B*) Typical examples of evoked action potentials *(A*) and recordings of spontaneous postsynaptic currents *(B*) obtained from layer 2/3 pyramidal neurons in the somatosensory cortex of male adult high and low LG rats. (*C,D*) Normalized cumulative frequency (*C*) and cumulative amplitude *(D*) of spontaneous events recorded from layer 2/3 pyramidal neurons. The amplitude distribution of high LG rats is shifted leftwards as compared to low LG rats (p<0.01, Kolgomorov-Smirnov test), whereas the frequency distributions do not differ significantly.

The morphological changes in hippocampus were accompanied by an increase in synaptic functioning in high LG rats [12]. Several studies have found that changes in dendritic morphology can have a major influence on neuronal firing and synaptic transmission [26]–[28]. In our study, we could not detect differences in the firing of layer 2/3 pyramidal neurons between high and low LG rats (Figure 2A). Also postsynaptic glutamate receptor expression levels, which have been shown to be under influence of maternal care, are of importance in synaptic functioning [4]. In the current study, we recorded spontaneous postsynaptic currents in layer 2/3 pyramidal neurons (Figure 2B) and found that the amplitude of the recorded events was decreased in high relative to low LG rats (p = 0.005, Kolgomorov-Smirnov test, high LG *n* = 10, low LG *n* = 8, Figure 2D). The frequency of spontaneous events tended to be lower in high relative to low LG rats, but this did not reach significance (Figure 2C). The input resistance of layer 2/3 pyramidal neurons (Figure 2A) amounted to 137.2±22.6 MΩ (*n* = 10) in high LG rats and 161.3±38.3 MΩ (*n* = 8) in low LG rats but was also not significantly different (p = 0.579). Taken together, the functional data indicate that a more slender phenotype of the dendritic tree and a decrease in dendritic spine density in high LG rats do not affect neuronal firing patterns. The observed changes in amplitude of the synaptic events could be an indication that also in the cortex maternal care modulates postsynaptic glutamate receptor densities resulting in changes in synaptic functioning.

An increasing number of studies have proposed that a substantial part of the effects of maternal care are mediated via epigenetic modulation of gene expression [7]–[9]. However, the molecular mechanism underlying the differences in dendritic development and function in high and low LG offspring is not known. Using microarray and real-time PCR analysis, it has previously been reported that maternal care affects the expression of a variety of genes in the hippocampus, including the glycoprotein reelin which was shown to be upregulated in high relative to low LG rats [17]. Reelin plays a pivotal role in neuronal development and function [15], [16]. In the hippocampus, it has been shown that via the VLDL/ApoER2 signal transduction pathway reelin regulates dendritic development and interferes with NMDA receptor expression and activity thereby affecting synaptic input [29]–[33]. Recently, we added to these findings that in the postnatal cortex, reelin regulates dendritic arborization of cortical layer 2/3 pyramidal neurons [18]. In addition, it has been found that the reelin promoter is highly sensitive to epigenetic modulation via histone acetylation and DNA demethylation [13], [14].

**Figure 3:**
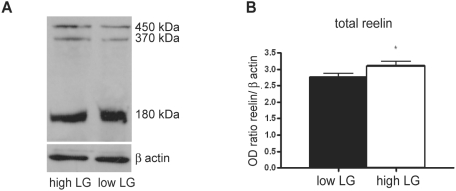
Differential reelin levels in the cortex of adult high and low LG rats. (*A*) Western blots immunoprobed for reelin with three bands at 450, 370 and 180 kDA corresponding to the full length and truncated reelin fragments in high and low LG rats. (*B*) Reelin immunoreactivity expressed as a ratio in optical density for reelin/ β actin for the accumulative reelin bands from high LG and low LG rats. Data are expressed as the mean±SEM. Asterisks indicate significant differences (* p<0.05).

Here we analysed the protein levels of reelin in the cortex using Western blot. Three typical reelin immunoreactive bands of 450 kDa, 370 kDa and 180 kDa with different densities corresponding to the full length and truncated reelin fragments can be distinguished, in agreement with the study of Jossin et al. [34] (Figure 3A). The optical density derived from the cumulative reelin bands was significantly higher in high relative to low LG offspring (p = 0.027, *n* = 16, Figure 3B). Taken together, the results indicate that differential maternal care affects the expression of reelin in the neocortex in a (qualitative) similar way (i.e. increase levels) as previously observed in the hippocampus [17].

The previously published data on the possible involvement of reelin in dendritic development [18], [33] and synaptic functioning [30]–[32] combined with our present observation that dendritic development and function are differentially regulated by maternal care in neocortex (Figure 1) as compared to hippocampus [12] suggests that reelin plays a dual role in the regulation of dendritic morphology in hippocampus and neocortex. However, we cannot exclude the possibility that not reelin but other factors are involved in the epigenetic regulation of dendritic development. It is of interest to note that reelin, apart from acting on its canonical receptors VLDL/ApoER2, can also activate integrin receptors and mediate activity-regulated cytoskeletal (Arc) protein synthesis [35], [36] which may be involved in dendritogenesis. Differential expression of receptor-signal transduction pathways, activated by reelin, in hippocampus and neocortex could therefore possibly underlie the presently observed differential effects of maternal care on dendritic morphology.

## Methods

### Ethics Statement

Experiments were performed according to the guidelines of the ethical committees of the Universities of Leiden and Amsterdam.

### Animals

The material used in this study originates from the same cohort of animals which was used in a previous study [12]. Briefly, Long Evans rats were bred in our colony at Leiden University, Netherlands. Maternal care characterization was performed in accordance with the method previously described by Meaney's group [37]. From weaning (PND 21), male offspring of low and high LG mothers were group-housed (4 per cage) with their respective littermates, with access to food and water *ad libitum* on a 12h/12h light dark cycle. Low and high LG offspring used in the current study were between 2–3 months of age.

### Quantitative Morphological Analysis: Golgi-Cox Method

Adult rat offspring (low LG *n* = 7, high LG *n* = 8) were used for morphological analysis of pyramidal neurons of layer 2/3 of the somatosensory cortex using the Golgi-Cox method as described previously [12]. Briefly, brains were removed and placed in vials containing Golgi-Cox solution and stored in the dark for 30 days. Brains were rinsed in milli-Q water, dehydrated in ethanol and embedded in celloidine and immersed in chloroform for a maximum of 16 hours. Chloroform was discarded and brains were subsequently immersed in 70% ethanol. Brains were sliced on a vibratome at a thickness of 100 µm. Sections were stained using procedures described before and mounted on glass slides. Slides were allowed to dry for two weeks before initiating the image analysis.

The complexity of apical and basal dendrites was determined from 27 and 28 cells taken from high LG and low LG offspring, respectively. The cells were randomly chosen, alternating between right and left hemisphere, in the somatosensory cortex. Analyses were performed by individuals unaware of the animal's phenotype, using a combination of Neurodraw and Image Pro software as described before [12]. The DCI was calculated as previously described [19]. Briefly, we assigned each branch tip an order value that equaled the number of branch points between the tip and the base of its primary dendrite. The DCI was subsequently calculated according to:
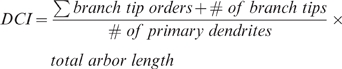
Spine density was determined on dendritic segments at fixed distances from the soma. Spines were counted in two 20 µm segments from the apical dendrite (90–110 µm and 190–210 µm distant from the soma) and one 20 µM segment from the basal dendrite (40–60 µm distant from the soma) for each cell. The length of the spine as well as subtypes of spines (e.g. stubby, thin or mushroom spine head, and filopodia) were scored according to Harris et al. [20]. Analysis was performed while being unaware of the animal's phenotype, using a combination of Neurodraw and Image Pro software.

### Electrophysiology

Five animals per group were sacrificed by decapitation, and after dissection of the brain, 300 µm thick coronal slices were cut on a vibroslicer (Leica VT1000S) in ice cold modified ACSF containing (in mM): CholineCl (120), KCl (3.5), CaCl_2_ (0.5), MgSO_4_ (6), NaH_2_PO_4_ (1.25), NaHCO_3_ (25) and glucose (25) continuously bubbled with carbogen (95% O_2_ and 5% CO_2_), pH = 7.4. During recording, slices were kept submerged at room temperature and continuously superfused with ACSF containing (in mM): NaCl (120), KCl (3.5), CaCl_2_ (2.5), MgSO_4_ (1.3), NaH_2_PO_4_ (1.25), NaHCO_3_ (25) and glucose(25), continuously bubbled with carbogen (95% O_2_ and 5% CO_2_) pH 7.4. Cortical layer 2/3 pyramidal neurons were visualized using differential interference contrast videomicroscopy on a Zeiss FS2 microscope. Patch pipettes were pulled from boroscilate glass and had a resistance of 3.5–5.5 MΩ when filled with internal solution containing (in mM): Kgluconate (110), KCl (30), CaCl_2_ (0.5), EGTA (5), HEPES (10), Mg-ATP (2), pH = 7.3 with KOH. Whole cell recordings were made using an EPC9 patchclamp amplifier and PULSE software (HEKA Electronic GmbH, Germany). Signals were filtered at 1–5 kHz and sampled at 2–10 kHz. Series resistance ranged from 7–21 MΩ and was compensated for approximately 60%. Cells were voltage clamped at −65 mV. Spontaneous events were recorded for 5 min. Data were analyzed with custom made software using Igor Pro (Wavematrics, Portland, OR) as described previously [38].

### Western Blotting

The brains of four adult rats per group were quickly extracted and collected in ice-cold homogenization buffer containing in mM HEPES (10), sucrose (320) and the EDTA free protease inhibitor cocktail (Roche, The Netherlands) pH = 7.4 with KOH. The frontal brain was dissected and homogenized on ice with a pestle in a 1 ml glass tube. The homogenate was centrifuged at 800 rpm for 10 min at 4°C, and the supernatant was centrifuged again at 15.000 rpm for 60 min at 4°C. The supernatant was stored at −80°C. The amount of protein was estimated via the Bradford method using a BCA protein assay reagent kit (Pierce, Rockford, IL). Samples of 20 µg were loaded in quadruplicate and proteins were separated by SDS-PAGE using 5% gels with 3% stacking gel on top of it, runned at 150 V for 60 min and rinsed in transferbuffer. Subsequently, proteins were transferred to nitrocellulose membranes and ran at 10 V for 30 min. Dry membrane was treated with blocking solution in TBST (0.05% Tween 20) containing 4% skim milk, incubated in 1∶1000 G10 anti-reelin antibody (Abcam, UK) in blocking solution for 2 hours, rinsed with TBST, incubated in 1∶1000 HRP conjugated goat anti-mouse IgG (Biorad, Hercules, CA) in blocking solution for 1 hour, rinsed with TBST and visualized using a chemiluminescence detection system (ECL kit and HCL hyperfilm, Amersham bioscience, UK). Optical density measurements were normalized using β-actin to control for the amount of protein loaded on the gel. Therefore, membrane was stripped with stripping buffer containing in 100 mM glycine pH 2.5 with HCl for 30 min. Subsequently, membrane was rinsed in TBST and and reprobed with a β-actin antibody 1∶2000 (Sigma, The Netherlands). β-actin immunoreactive bands were visualized according the procedure described above. After development of the film, the image was scanned and the optical density of the bands was determined using the ImageJ software and Gel analyzer plug-in software.

### Statistical Analysis

Results of quantative morphological analysis for dendritic complexity and spine density, average amplitude and frequency of electrophysiological recordings and Western blot measurements were considered significantly different if p<0.05, using a two-tailed Student's t-test, with all data expressed as mean±S.E.M. Kolgomorov-Smirnov test was used to compare the normalized cumulative amplitude- and frequency distributions of the spontaneous activity.
